# Geostatistical analysis and mapping of malaria risk in children under 5 using point-referenced prevalence data in Ghana

**DOI:** 10.1186/s12936-019-2709-y

**Published:** 2019-03-11

**Authors:** Robert Yankson, Evelyn Arthur Anto, Michael Give Chipeta

**Affiliations:** 1 African Institute of Mathematical Sciences, Accra-Cape Coast Road, Adisadel, Cape Coast, Ghana; 2 Malawi-Liverpool Wellcome Trust Research Programme, Queen Elizabeth Central Hospital, Blantyre, Malawi

**Keywords:** Malaria, Hotspot, Mapping, Geostatistics, Exceedance probability

## Abstract

**Background:**

Malaria remains a major challenge in sub-Saharan Africa and Ghana is not an exception. Effective malaria transmission control requires evidence-based targeting and utilization of resources. Disease risk mapping provides an effective and efficient tool for monitoring transmission and control efforts. The aim of this study is to analyse and map malaria risk in children under 5 years old, with the ultimate goal of identifying areas where control efforts can be targeted.

**Methods:**

Data collected from the 2016 Ghana demographic and health survey was analyzed. Binomial logistic regression was applied to examine the determinants of malaria risk among children. Model-based geostatistical methods were applied to analyze, predict and map malaria prevalence.

**Results:**

There is a significant association of malaria prevalence with area of residence (rural/urban), age, indoor residual spray use, social economic status and mother’s education level. Overall, parasitaemia prevalence among children under 5 years old for the year 2016 is low albeit characterized by “hotspots” in specific areas.

**Conclusion:**

The risk maps indicate the spatial heterogeneity of malaria prevalence. The high resolution maps can serve as an effective tool in the identification of locations that require targeted interventions by programme implementers; this is key and relevant for reducing malaria burden in Ghana.

## Background

The recent world malaria report estimated that 216 million cases of malaria and 445,000 deaths occurred worldwide in 2016; the number of cases increased by approximately 5 million compared to the previous year [1]. Malaria burden is greatest in sub-Saharan Africa (SSA) where an estimated 90% of all malaria deaths occur, and children under 5 years old account for 78% of all deaths [2]. The World Health Organization (WHO) estimated that one child in SSA dies from malaria every 2 minutes [3].

Malaria is a major threat to public health and a leading cause of morbidity and mortality especially among children under 5 years old in Ghana [4, 5], with prevalence estimated at 21% as of 2016 [6]. Approximately 20,000 children die from malaria every year in Ghana, 25% of whom are children under 5 years old. Malaria has been shown to be intimately connected to poverty; it is both a root cause and a consequence of poverty, such that the burden is most intractable in communities and countries that are the most poorest [7]. Ill-health in poor settings leads to reduced ability of people to deal with the disease burden. Malaria burden exerts a negative impact on economic productivity due to human development and financial burden on the economy overall, and on the affected households specifically [8, 9]. It is estimated that a single episode of malaria in Ghana results in an average loss of 5 workdays; 3 days for the patient and 2 days for the caretaker [10].

Evidence shows that malaria in children under 5 years can be attributed to a number of factors including not using insecticide-treated bed nets (ITNs), not sleeping in indoor residual sprayed (IRS) rooms, age of the child and lack of timely diagnosis of suspected cases, among others [5, 8, 11]. The Ghana Health Service (GHS) has set an ultimate goal of reducing malaria morbidity and mortality by 75% (using 2012 as baseline) by the year 2020, through various integrated control programmes [12]. The national malaria control programme (NMCP) has been scaling up various malaria control interventions throughout the country [13]. These include vector monitoring and control, use of long-lasting insecticidal nets (LLIN), intermittent preventive treatment in pregnancy (IPTp), effective case management, and social and behaviour change communication (campaign on test, treat and track) [12]. These efforts need to be targeted to areas where they would have most health impact.

Malaria transmission in Ghana is driven mainly by two main vectors namely *Anopheles gambiae* (sensu lato) and *Anopheles funestus*. Their peak activities occur at the end of the wet season [14]. Like many malaria endemic countries in SSA, malaria transmission in Ghana is highly heterogeneous both spatially and temporally [15, 16]. The levels of transmission intensity in space and time are significantly linked to changes in climate, altitude, topography, land use/human settlement and environmental factors [17] among others; these factors profoundly influence the vector, and hence the parasite and transmission patterns. The southern (forest and coastal ecological zones) part of Ghana has transmission almost all year round while the northern (savannah ecological zones) part usually experiences seasonal transmission in the wet season [18–20]. Knowledge of the local spatial and temporal heterogeneity of malaria transmission is essential for the planning and evaluation of malaria interventions [21]. This justifies the timely identification of locations requiring targeted interventions to optimize usage of resources in resource-limited settings.

A growing emphasis is now being placed on the need to timely identify sub-national variation and areas that lag behind in performance of malaria control and prevention despite the current increasing efforts to curb the burden [22–24]. Risk mapping provides an effective and efficient tool for disease monitoring and control [24, 25]. In the past, several methods have been adopted in producing malaria risk maps, including theoretical climatic models, reservoir or vector surveys and expert opinion [26]. In 2013, the Ghana NMCP together with its partners presented a comprehensive epidemiological profile for malaria risk in children aged 2–10 years. This nationwide study reported national- and district- level estimates [27]. Kumi-Boateng et al. [16] presented a spatial multi-criteria decision analysis (MCDA) incorporated into geographic information system (GIS), where they mapped the effect of several covariates on endemicity of malaria prevalence in Ghana. In a different study, Kumi-Boateng et al. [28] used GIS, satellite remote sensing (RS) and analytical hierarchy process to develop malaria risk map for New Juaben municipality in the Eastern region of Ghana. These studies provide estimates at either district, regional or national level. Meanwhile, the global malaria elimination programme classifies Ghana and much of West Africa among nations considered to be in the control phase [1]. Despite these recent positive outcomes, some areas are still lagging behind in performance and need urgent identification for targeted interventions. This calls for risk mapping at finer scales than those reported previously.

In this study, spatial patterns of malaria prevalence in children under 5 years were mapped at a fine-scale of 5 × 5 km resolution in order to identify “hotspots” (i.e. geographic areas where malaria prevalence is above average or some threshold) using the model-based geostatistical (MBG) techniques. Geostatistical techniques and models are increasingly finding their application in the analysis and mapping of malaria incidence and prevalence, among other fields. The methods permit simultaneous modeling of related issues such as risk assessment, spatial dependence, prediction and quantification of uncertainty [29, 30]. Geostatistical methods provide a feasible and statistically principled approach to model spatio–temporally referenced survey data from low-resource settings [31]. The fine-scale risk maps produced will enable the Ghana NMCP to identify areas that can be targeted with health interventions in order to have the most health impact. It is thought that strengthened control in malaria hotspot areas is imperative to efficiently achieve malaria elimination [32–35]. Previous research has shown that reducing transmission in hotspots may reduce transmission in the wider community [36, 37] and, therefore, a more cost-effective means to approach elimination [33], and may ethically justify “unequal, but equitable,” allocation of resources [38].

## Materials and methods

### Study area, design and sample

**Fig. 1:**
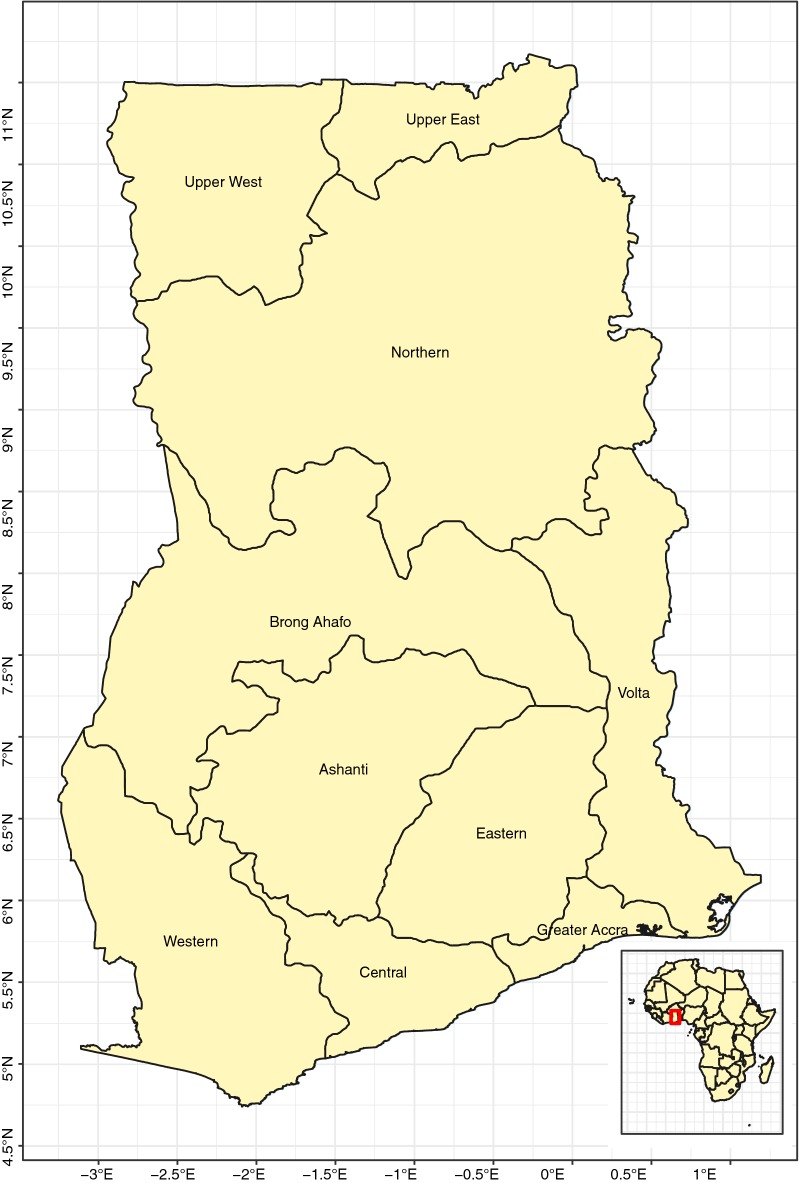
The 10 administrative regions covering the study area for Ghana demographic health survey

Ghana is located between latitudes $4^{\circ }$ and $12^{\circ }$N and longitudes $4^{\circ }$W and $2^{\circ }$E, see Fig. 1. Geographically, Ghana is closer to the centre of the world than any other country, and the Greenwich Meridian passes through the country. Ghana has a total land area of 238,538 km${^2}$; with 840 km distance from South to North and 554 km from East to West. Ghana shares border with Togo to the East, Cote d’Ivoire to the West, Burkina Faso to the North and has a coastline in the South along the Gulf of Guinea. There are 10 administrative regions in Ghana [6].

In this study, data from the Ghana malaria indicator survey (MIS) were used; malaria indicator surveys are conducted as part of the demographic and health surveys (DHS) and are well known [6, 39, 40]. The 2016 Ghana DHS was conducted through the malaria indicator survey (MIS) implemented by the Ghana Statistical Service (GSS), in collaboration with the Ghana NMCP and the national public health reference laboratory (NPHRL) of the GHS [6]. The 2016 Ghana MIS collected information that complements routine administrative data which are used to inform strategic planning and evaluation of Ghana’s malaria control programme [6]. Information on malaria prevention, treatment and prevalence were obtained during the survey. Specifically, data were collected on key malaria indicators such as the proportion of the ownership and use of ITNs, assessment of the coverage of IPT to protect pregnant women against malaria, estimated prevalence of malaria and anaemia among children aged 6–59 months, IRS coverage, wealth index of households, region where the survey took place, area of residence, gender, children’s age in months, mother’s education and others [6]. The response variable is the outcome of malaria test.

The 2016 Ghana MIS was designed and conducted to provide estimates of key malaria indicators for the whole country, for urban and rural areas separately, and for each of the ten administrative regions [6]. The administrative regions are Ashanti, Brong Ahafo, Central, Eastern, Greater Accra, Northern, Upper East, Upper West, Volta and Western. The sample frame used was the 2010 population and housing census (PHC), using a complete list of enumeration areas (EA). In this two-stage design, the first stage was to select 200 EAs with probability proportional to EA size. In the second stage, a fixed number of 30 households were then sampled from each of the selected EAs, giving a nationally representative sample of 6000 households.

Ghana MIS collected data via both the computer-assisted personal interviewing (CAPI) system on tablet computers and paper-based questionnaires. The three main questionnaire types used were the household questionnaire, the woman’s questionnaire and the biomarker questionnaire [6]. Blood samples for malaria testing were collected by finger- or heel-prick from children aged 6–59 months, and then tested for malaria with SD bioline malaria *Ag P.f./pan* rapid diagnostic test (RDT). Malaria RDT results were recorded in the biomarker questionnaire and the result shared with the child’s parent or guardian [6]. Additionally, microscopy results were read in the laboratory. Children who tested positive or showed signs and symptoms for malaria were either offered a full course of medication according to standard treatment protocol in Ghana or were referred to a health facility [6]. In the current analysis, only RDT tests results were used.

### Statistical analysis

The formulation of the geostatistical model in the current study follows from the standard geostatistical model for prevalence surveys in Diggle et al. [41]. Consider a field team that visit communities at different locations $x_i$ in a study region say $G \subset \mathbb {R}^2$, and sample $m_i: i = 1,\dots ,n$ individuals at risk in each community, as well as taking the records of whether each individual tests positive or negative for the disease under study. Let $Y_i$ denote the number of positive malaria RDT outcomes out of $m_i$ individuals tested at locations $x_i$ in a region of interest $G \subset \mathbb {R}^2$, and a vector of associated covariates $\varvec{d(x_i)} \in \mathbb {R}^p$. The standard geostatistical model then assumes that $Y_i \sim \textit{Binomial} (m_i, p(x_i))$, where $p(x_i)$ measures the disease prevalence at locations $x_i$. Adopting the logistic link function, the model further assumes that:

1 $$\begin{aligned} \log \Big \lbrace \frac{p(x)}{\lbrace 1 - p(x)\rbrace }\Big \rbrace = \alpha + \varvec{d(x)^{\prime }\beta } + S(x) \end{aligned}$$

where $\alpha$ is the intercept parameter, *S*(*x*) is an unobservable random effect which is Gaussian process with zero-mean and a constant variance $\sigma ^2$; $d(\cdot )$ represents a vector of observed spatial explanatory variables associated with the response *Y*,  and $\varvec{\beta }$ is a vector of spatial regression coefficients for the covariates. The empirical logit transform is defined as follows:

2 $$\begin{aligned} Y_{ij}^* = \log \Big \lbrace \frac{(Y_{ij} + 0.5)}{(m_{ij} - Y_{ij} + 0.5)}\Big \rbrace \end{aligned}$$

and the underlining assumption is that:

3 $$\begin{aligned} Y_{ij}^* = \alpha + \varvec{d(x_{ij})}^{\prime }\beta + S(x_{i}) + Z_i, \end{aligned}$$

where $Z_i$ are mutually independent zero-mean Gaussian random variables with variance $\tau ^2$. The index *i* represents the household and the index *j* represents an individual within the household. The transformation in Eq. (2) was preferred here as it allows for a computationally simpler non-hierarchical approximate model fitting [42], this is especially advantageous where computational resources are limited.

Throughout the analysis, a Matérn parametric family of correlation functions for the process *S*(*x*) is assumed, to enable the definition of a legitimate class of covariance functions for the data. The Matérn correlation function is positive definite and sufficiently flexible [30]. The process *S*(*x*) is assumed to have mean of zero, stationary and isotropic Gaussian process, that is, with invariant distribution under translation and rotation [43]. The Matérn correlation function [44] for stationary Gaussian processes is a two-parameter family given by:

4 $$\begin{aligned} \rho (u,\phi ,\kappa ) = \lbrace 2^{\kappa -1}\Gamma (\kappa )\rbrace ^{-1}(u/\phi )^{\kappa } K_\kappa (u/\phi ), \end{aligned}$$

in which *u* denotes the distance between two locations *x* and $x^{\prime }$, $\phi > 0$ is a scale parameter that determines the rate at which correlation decays to 0 as the distance increases, and $\kappa > 0$, is a shape parameter which determines the analytic smoothness of the underlying process *S*(*x*). Also, $\Gamma (\kappa )$ denotes the smallest integer greater than or equal to $\kappa$, and $K_{\kappa }(\cdot )$ denotes a modified Bessel function of order $\kappa > 0$ [30].

A non-spatial generalized linear model (GLM) was fitted in the first step. For variable selection in the GLM, ordinary binomial logistic regression model was used, retaining covariates with nominal* p*-values less than 0.05. The resulting covariates are presented in Table 2 with terms for residence, age, indoor residual spray use, wealth index and mother’s education. In the second step, a spatial model was fitted. The Matérn shape parameter $\kappa$ and relative variance parameters $\tau ^2$ were fixed at 1.5 and 0, respectively.

In the spatial analysis, estimates of the model parameters were obtained and used to make spatial predictions over a fine grid of 5 $\times$ 5 km, over the whole of Ghana. Under a predefined control scenario, malaria risk of children aged 24–36 months, middle household wealth status, middle mothers’ education level, with no indoor residual status and assumed rural residence for all the unsampled locations, was mapped. All the analysis and mapping were carried out using the R statistical software environment version 3.5.0 [45].

### Policy relevant criteria for interventions

One of the objectives of the current study is to identify “hotspots” (here defined as areas that are above a prevalence threshold, say *t*). In prevalence estimation analyses, it is worthy noting that the resulting estimates *p*(*x*) at a location *x* have uncertainty that needs to be taken into account. It has been shown that classifying areas into different endemic levels purely based on estimates of *p*(*x*) at location *x* can lead to unwarranted policy decisions [31]. To overcome this issue, the geostatistical model developed in the statistical analysis section above, was used to derive a distribution of the most likely values that *p*(*x*) can take. This distribution was then used to quantify how likely *p*(*x*) is to be above a threshold *t* through the so-called *exceedance probability* (EP), formally expressed as:

5 $$\begin{aligned} EP = Probability\{p(x) > t | data\} \end{aligned}$$

where *t* is the prevalence threshold, set to 20% in the current analysis. In other words, EP expresses how likely prevalence is to be above the threshold *t* based on the available survey data. An EP close to 100% indicates that prevalence is highly likely to be above the threshold *t*; if close to 0%, prevalence is highly likely to be below the threshold *t*; finally, if close to 50%, prevalence, is equally likely to be above or below the threshold *t*, hence this corresponds to the highest level of uncertainty. This is important when defining the level of certainty that a locality is above 20% and might be considered suitable for targeted interventions or proves that an area is highly intractable to interventions applied in that area to-date. It is desirable to be $\ge$ 80% or $\ge$ 90% certain that this is a real value based on the available data. If a locality does not reach the required level of certainty, additional sampling effort or surveys are required in order to classify that into the appropriate endemic level.

In some cases, the interest may be in delineating areas where *p*(*x*) is less than the threshold *t*, that is, areas that are below a prevalence threshold, through *non-exceedance probability* (NEPs), formally expressed as:

6 $$\begin{aligned} NEP = Probability\{ p(x) < t | data\} \end{aligned}$$

NEP expresses how likely *p*(*x*) is below the threshold *t* based on the available survey data. A NEP close to 100% indicates that *p*(*x*) is highly likely to be below the threshold *t*; if close to 0%, *p*(*x*) is highly likely to be above the threshold *t*; finally, if close to 50%, *p*(*x*) is equally likely to be above or below the threshold *t*, hence this corresponds to the highest level of uncertainty. Here, areas that have 20% or less malaria prevalence based on the available 2016 GDHS data are shown, with 80% and 90% certainty. The 20% threshold was chosen based on the prevailing malaria prevalence in Ghana, which was 21% as per the MIS in 2016 [6]

### Model validation

In order to justify the need for modeling using the spatial geostatistical model applied here, evidence against the residual spatial correlation in the data was tested using the following 5-step variogram-based validation algorithm [31, 46].

1. Generate a point estimate of $Z(x_i)$ i.e. ${\widetilde{Z}}(x_i)$ from a non-spatial model, for each observed location $x_i$. This model assumes the absence of any residual spatial correlation such that $S(x) = 0 \,\forall$ *x*;
2. Permute the order of the data, including ${\widetilde{Z}}(x_i)$, while holding $(x_i)$ fixed;
3. Compute the empirical semi-variogram for ${\widetilde{Z}}(x_i)$;
4. Repeat steps (1) and (2) a large number of times, ***T***, say ***T*** = 1000;
5. Use the resulting ***T*** empirical variograms to generate 95% confidence intervals at each of the pre-defined distance bins.

To conclude that there is no evidence against the adopted spatial model correlation, the empirical semi-variogram from the original data must fall within the generated 95% confidence intervals.

## Results

There were a total of 2537 children in the dataset. Table 1 summarizes the proportions of children with a positive malaria outcome. Overall, malaria prevalence among children under 5 years in Ghana for 2016 was estimated at 22.1%. Prevalence was shown to increase with child’s age, younger children have the lowest prevalence at 16% among children below 12 months, and older children showing the highest prevalence at 28% among children between 48 and 59 months. Minor difference in prevalence were observed between males and females, with males having a slightly higher prevalence at 23% compared to 22% for females.

Children of mothers with no education were shown to have the highest malaria prevalence at 31% compared to children of mothers with higher education level (6%). A general trend observed showed that increasing levels of education were associated with decreasing malaria prevalence in children. Children from the poorest households had the highest proportion of a positive malaria outcome. Table 1 shows that the proportion of malaria prevalence decreases with an increase in the wealth status of households. Children from the poorest households recorded the highest proportion of 32%, whilst those from the highest wealth status recorded the lowest proportion at only 3%. This may be an indication that children from well-endowed households are less prone to malaria than those from poor households. Children from households that did not receive IRS treatment had a high parasitaemia prevalence at 23% as compared to 19% of children from IRS treated households.

**Table 1:** Proportions of malaria among children aged under 5 years with respect to covariates under consideration

	Total	RDT	
		Negative	Positive
Age (in months)			
$<12$	289 (11%)	244 (84%)	45 (16%)
12–23	602 (24%)	490 (81%)	112 (19%)
24–35	588 (23%)	454 (77%)	134 (23%)
36–47	551 (22%)	421 (76%)	130 (24%)
48–59	507 (20%)	367 (72%)	140 (28%)
Gender			
Male	1293 (51%)	1002 (77%)	291 (23%)
Female	1244 (49%)	974 (78%)	270 (22%)
Mothers educ.			
No education	876 (35%)	605 (69%)	271 (31%)
Primary	509 (20%)	398 (78%)	111 (22%)
Middle	798 (31%)	644 (81%)	154 (19%)
Secondary	228 (9%)	210 (92%)	18 (8%)
Higher	126 (5%)	119 (94%)	7 (6%)
Wealth status			
Lowest	892 (35%)	605 (68%)	287 (32%)
Lower	486 (19%)	344 (71%)	142 (29%)
Middle	411 (16%)	342 (83%)	69 (17%)
Higher	413 (16%)	360 (87%)	53 (13%)
Highest	335 (13%)	325 (97%)	10 (3%)
IRS			
No	2055 (81%)	1584 (77%)	471 (23%)
Yes	482 (19%)	392 (81%)	90 (19%)
Residence			
Urban	995 (39%)	884 (89%)	111 (11%)
Rural	1542 (61%)	1092 (71%)	450 (29%)
Region			
Ashanti	275 (11%)	237 (86%)	38 (14%)
Brong Ahafo	239 (9%)	187 (78%)	52 (22%)
Central	217 (9%)	152 (70%)	65 (30%)
Eastern	208 (8%)	143 (69%)	65 (31%)
Greater Accra	229 (9%)	219 (96%)	10 (4%)
Northern	431 (17%)	295 (68%)	136 (32%)
Upper East	289 (11%)	244 (84%)	45 (16%)
Upper West	228 (9%)	178 (78%)	50 (22%)
Volta	213 (8%)	164 (77%)	49 (23%)
Western	208 (8%)	157 (75%)	51 (25%)

Malaria prevalence was high in rural areas at 29% compared to 11% among children in urban settings. This result agrees with previous studies, for example, Nyarko and Cobblah [5] found that malaria prevalence was highest among children from rural settings compared to urban settings. At 32% prevalence, the Northern region had the highest under 5 malaria, this is followed by Central and Eastern regions, recording 30 and 31%, respectively. Greater Accra region had the lowest prevalence at 4%. It is worthy noting that the Greater Accra region is the most urbanized region in Ghana with 87.4% of its total population living in urban centers [47]. The climate of Ghana has a principal feature of alternate wet and dry seasons caused by the interaction of the Inter-Tropical Convergence Zone and the West African Monsoon [48]. The Southern Ghana; of which the Central, Eastern and Greater Accra regions are part, is characterized with two distinct wet seasons, while Northern Ghana has only one wet season that begins in May and ends in October. In the Southern Ghana, the first rainy season is from May to June, with the heaviest rainfall occurring in June while the second rainy season is from September to October [48].

### Model results

The response variable for each child was the binary outcome of the test for presence/absence of malaria from a finger- or heel-prick blood sample. Results from a GLM model are presented in Table 2. The binomial logistic model in Eq. (1) was fitted to obtain the Monte Carlo maximum likelihood [49] estimates of the parameters and associated 95% confidence intervals, as shown in Table 2. The $\sigma ^2$ and $\phi$ are variance of the Gaussian process and scale of the spatial correlation, respectively.

**Table 2:** Monte Carlo maximum likelihood estimates and 95% confidence intervals for the binomial logistic model fitted to 2016 GMIS under 5 malaria prevalence data

Term	Estimate	95% confidence interval
Intercept	− 1.733	(− 2.625, − 0.841)
Residence (R)	0.473	(0.095, 0.852)
Age	0.212	(0.128, 0.297)
IRS	− 0.239	(− 0.703, 0.226)
Wealth index	− 0.357	(− 0.500, -0.214)
Mothers educ.	− 0.145	(− 0.276, -0.015)
$\sigma ^2$	0.983	(0.652, 1.480)
$\phi$	12.819	(8.043, 20.430)

The scale parameter $\phi$ has units in kilometres

**Fig. 2:**
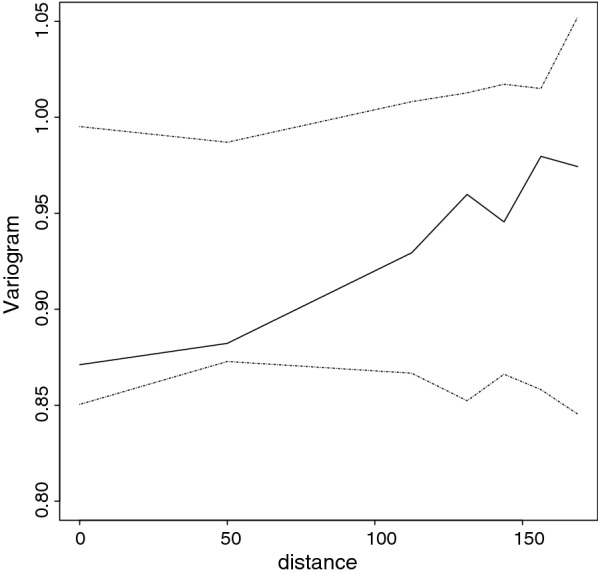
Model validation plot, the solid line is the variogram based on the residuals from a non-spatial model (empirical semi variogram). The dashed lines are the 95% confidence intervals generated under the fitted spatial model

The validity of the adopted spatial structure used in the modeling exercise was tested using steps outlined in the model validation sub-section above. This is important especially when identifying areas where prevalence lies below (i.e. NEP) or above (i.e. EP) pre-defined thresholds. The results of this process are shown in Fig. 2. Since the empirical semi-variogram (solid line) falls within the 95% confidence intervals (dashed lines), then the adopted covariance model is compatible with the malaria parasite prevalence data implying that the results of NEPs, EP and the model overall are valid.

From Table 2, residing in the rural areas and child’s age are associated with an increase in probability of a positive malaria outcome in children. Household wealth status and mother’s education are negatively associated with the probability of a positive outcome, whereas IRS shows a negative, but non-significant association.

**Fig. 3:**
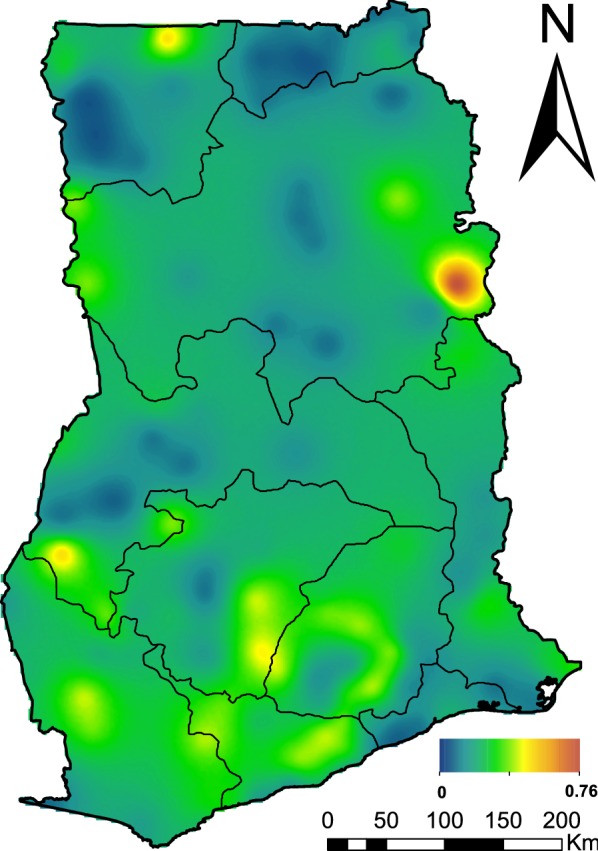
Malaria prevalence predictions among children aged under 5 years in Ghana

The 5 $\times$ 5 km resolution maps for malaria prevalence in children under 5 years are presented. Overall, prevalence is low at national level, with an average of 22% but characterized by areas that are above average prevalence. Hotspots were observed to be mainly localized in the Northern, Upper West, Western, Eastern and Central regions. The region with the highest prevalence (deep orange) is the Northern region, particularly the communities surrounding the Mo and Oti rivers. The map of predicted malaria prevalence is shown in Fig. 3.

**Fig. 4:**
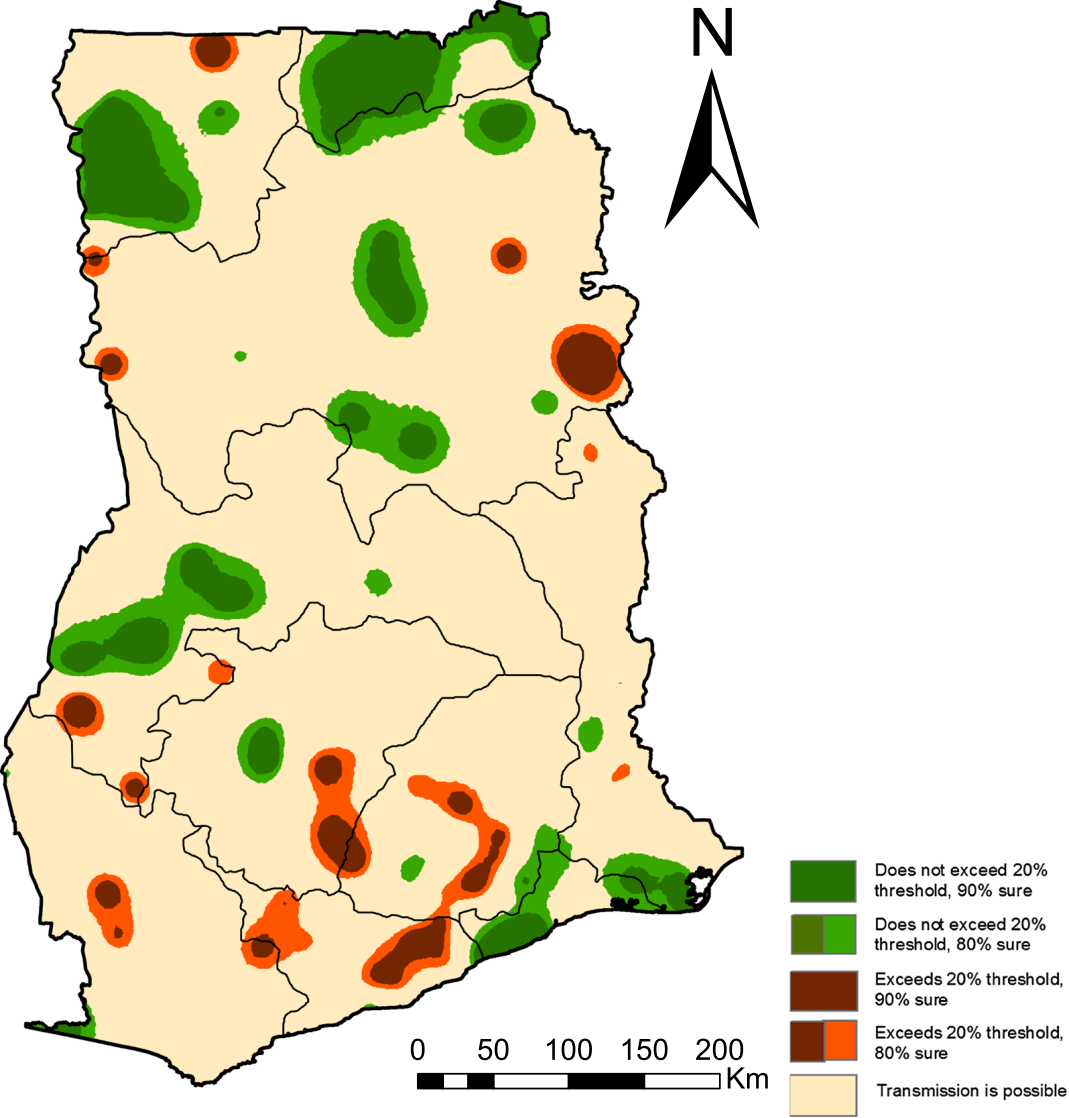
Map showing areas where transmission is above or below 20% threshold. Exceedance ($\ge$ 20%) and non-exceedance ($> 20 \%$) probabilities in Ghana in 2016

Figure 4 presents maps of malaria exceedance and non-exceedance probabilities, showing areas where $p(x) \ge 0.2 \mid data$ as well as areas where $p(x) < 0.2 \mid data$, with 80% and 90% certainty in both cases. Several regions, including, South western, Central, Upper West, Western, Eastern, Northern, and Ashanti have locations with predicted prevalence above 20%. The dark red areas show locations where prevalence is above 20%, at 90% certainty and light red are all areas where prevalence is above 20% at 80% certainty. In the same Fig. 4, non-exceedance probabilities are presented, showing areas where prevalence is less than 20% with 80% and 90% certainty. The following regions: Upper East, Greater Accra, Volta and Brong Ahafo have locations where predicted malaria prevalence in children under 5 years is less than 20%.

## Discussion

Malaria is a leading cause of death in most of SSA, especially among children under 5 years of age. Malaria monitoring and control programmes can benefit from the availability of accurate prevalence maps. Model-based geostatistical analysis in conjunction with active surveillance is an effective, practical strategy for producing accurate local-scale maps that can pick up hotspot areas in disease burden that can benefit immensely from targeted interventions. In this study, the Ghana 2016 MIS data were analyzed using MBG models to delineate and map areas where prevalence is above (or below) a given threshold, which could be policy relevant. The analysis also determined the risk factors on the geographical distribution of malaria prevalence in children under 5 years. Malaria prevalence prediction maps at a 5 $\times$ 5 km resolution show disease burden at one of the finest scale possible.

The MBG models fitted via Markov Chain maximum likelihood simulation methods were used to determine the adjusted effect of factors on malaria prevalence. Ordinary logistic regression was used for variable selection in order to determine and choose the most important predictors for explaining variation in malaria prevalence. Risk factors pertaining to malaria prevalence considered in the current study are area of residence (urban/rural), age of the child in months, indoor residual spraying, household wealth status and mother’s education level.

The association between area of residence and malaria prevalence is well known. Living in rural areas was positively associated with the probability of a child having a positive malaria outcome. Similar results were reported by Nyarko and Cobblah [5] who analyzed and reported data from the 2008 GDHS. This can be explained by a number of factors including access to facilities; people living in resource-limited rural settings tend to have lower access to health facilities as opposed to those living in urban settings. Malaria risk in urban areas is known to differ from those in rural areas, see, for example, Wilson et al., Hay et al. and Uzochukwu et al. [50–52]. Malaria is often referred to as a disease of poverty [53].

At the global level, malaria incidence has been shown to be concentrated in the world’s poorest countries, with 90% of malaria deaths occurring in SSA. In this region, majority of the population reside in the rural areas. This, therefore, has implications that the high prevalence of malaria in children from rural areas can be attributed to rural poverty, as compared to urban settings. Living in rural areas is associated with inadequate health services coupled with poor housing conditions which expose children to malaria transmitting vectors hence high malaria prevalence.

Older children were observed to be at an increased risk of being infected with malaria compared to infants. Similar outcomes have been observed in studies conducted in Tanzania and Uganda, see, for example, Hendriksen et al. [54] and Ssempiira et al. [55]. This can be explained by the fact that infants have immunity acquired from mothers, including passive transfer of antibodies through breastfeeding. With age, this immunity starts to wane hence children are at increased risk of malaria before they start to develop their own immunity following repeated infections [56–58].

It has been argued previously that age distribution of malaria cases is mostly influenced by malaria transmission intensity, severity and seasonality, especially in SSA [59, 60]. A review by Snow and Marsh [61] indicates that, in areas where transmission is very low, all age groups are likely to be infected with malaria (most likely in older children and adults due to occupational risk); in areas with moderate transmission intensity, older children and adults are less likely to be infected with malaria. In contrast, in areas with high transmission, the majority of malaria infection occurs in young children under one year of age. This supports the global malaria elimination programme classification, which classifies Ghana and much of West Africa among nations considered to be in the control phase [1].

Indoor residual spray use was associated with reduced malaria risk, albeit not significant. Meanwhile, IRS has previously been shown to significantly reduce malaria prevalence. Coleman et al. [62] showed that there was a significant decline in parity rates of vectors as a result of IRS in Northern Ghana [62–64]. The study observed a steady increase in parity rates following withdraw of IRS in the study area. Coleman et al. [62] further reported that these rates were high in areas where IRS was not applied. Similar results have been reported in Uganda by Robert and Matthew and Ssempiira et al. [11, 55] who observed a significant reduction in child’s malaria risk due to IRS. It is unsurprising to see a non-significant relation between IRS and malaria prevalence. In the sample available in the current study, only 18.99% of the population had IRS. A previous study has shown that there was no correlation between malaria prevalence and IRS due to low coverage of the latter, see, for example, Mumbengegwi et al. [65].

In the current study, it has been observed that household wealth status was negatively associated with malaria prevalence. Children living in wealthier households had a significantly lower malaria prevalence compared to children living in poorer households. This finding is consistent with findings from a study conducted in the Gambia. Sonko et al. [66] reported that children from the second, third, fourth and richest quintiles were significantly less likely to have malaria compared to children from the poorest quintiles. This can be explained in a number of ways, including that, highest wealth status households can afford malaria preventive measures, such as adequate housing facilities with screens that block vectors, insecticide-treated bed nets, quick diagnosis and acquiring of drugs in case of infection without depending on public facilities. Several studies have shown that malaria is highly correlated with poverty [67–69]. At a regional level, it has been shown that malaria burden is highest in the poorest countries, particularly in SSA where 90% of malaria deaths occur [1, 53, 66].

Education level of the child’s mother was shown to be highly associated with reduced malaria risk in children. Children whose mothers had no education at all were at an increased risk compared to those whose mothers had higher education. This result is similar to the results reported by Snyman et al., Robert and Mathew, Erhart et al. and Ssempiira et al. [11, 55, 70, 71], among others. In previous studies, higher education has been associated with better understanding of health issues generally. Again, it is assumed that mothers with higher education are more likely to have high socio-economic status, therefore being able to afford health care and preventive measures for malaria. Thus, the importance of education in malaria prevention cannot be overstated.

Malaria prevalence in children under 5 years is generally low at 22.1% in 2016 in Ghana, characterized by several hotspots. Model-based geostatistical methods allowed us to map prevalence at a fine-scale resolution of 5 $\times$ 5 km. Malaria transmission in Ghana is highly heterogeneous across space and time, peaking mostly in the wet season [15, 72]. High prevalence was observed in the Northern, parts of Upper West, Ashanti, Western, Central and some part of Brong Ahafo regions. Kumi-Boateng et al. [16] found a similar pattern, indicating a high prevalence in the central as well as the west-southern parts of Ghana. Figure 3 shows that most areas have low prevalence in general, except for a few locations with elevated risk, most notably in the Northern region within the communities surrounding the Mo and Oti rivers. One of the possible explanations for this could be that the rivers around these communities are supporting favorable conditions for the breeding of mosquitoes, hence increased transmission. In high transmission periods, hotspots tend to grow and fuel transmission; and they maintain transmission during low transmission periods [43, 73].

Model-based geostatistical methods are advantageous in low-resource settings where data are sparse in the sense that they enable estimation of disease risk at health decision-making units as well as properties of uncertainty. To follow up on this point, exceedance and non-exceedance probabilities were used to quantify uncertainty in estimates of malaria prevalence with respect to areas that are above or below a threshold of 20%. The importance of mapping these areas is that it allows focusing control efforts and the limited resources to areas where they would have maximum health impact. Figure 4 shows that most areas in the northern part of the country are well below a threshold of 20% prevalence, with 80% or 90% certainty. This implies that in these areas, a shift in control efforts towards pre-elimination can be considered. On the other hand, a number of localities in south-western and central regions have prevalence above 20%, both at 80% or 90% certainty. For programme implementers, control efforts in these areas would be different, instead, the focus would be on reducing transmission through preventive interventions such as mass bed-net distribution and/or indoor residual spraying campaigns. Thus, in the identified high transmission areas, control efforts would need to be more targeted and tailor-made as opposed to universal coverage effort, in order to cut transmission as much as possible.

The results presented here should be considered within the context of some limitations. First, only spatial analysis was carried out to show malaria prevalence heterogeneity in space. Malaria risk is known to be heterogeneous both in space and time, implying that the identified hotpots can potentially vary in size and location with season (time). Secondly, secondary data from Measure DHS’s malaria indicator survey database were used and analyzed. The database had limited variables that could have been included in the analysis to improve the understanding of malaria burden in children under 5 years in Ghana.

## Conclusion

The current study has shown that area of residence, child’s age, wealth status and mother’s education level are important risk factors for malaria prevalence in children under 5 years in Ghana. The fine-scale risk maps presented here show the contemporary under 5 children malaria situation in Ghana. The high resolution maps can be used for planning, implementation, resource mobilization, monitoring and evaluation of interventions in hotspots within the country. Thus, it is a useful tool for the GHS in reducing malaria morbidity and mortality by the targeted 75% by 2020, through integrated and targeted control measures. Fine-scale risk mapping is a relevant tool for all settings where there is a need for identifying hotspots in malaria endemic settings.
